# Geometry of the Carotid Artery and Its Association With Pathologic Changes in a Chinese Population

**DOI:** 10.3389/fphys.2019.01628

**Published:** 2020-01-21

**Authors:** Yiyao Cui, Xiaoshuo Lv, Feng Wang, Jie Kong, Hao Zhao, Zhidong Ye, Chaozeng Si, Lin Pan, Peng Liu, Jianyan Wen

**Affiliations:** ^1^Department of Cardiovascular Surgery, China-Japan Friendship Hospital, Beijing, China; ^2^Graduate School of Peking Union Medical College, Beijing, China; ^3^Department of Operations and Information Management, China-Japan Friendship Hospital, Beijing, China; ^4^Institute of Clinical Medical Science, China-Japan Friendship Hospital, Beijing, China

**Keywords:** carotid endarterectomy, carotid artery, geometry, carotid artery bifurcation angle, angiography, extracellular matrix, yes-associated protein

## Abstract

**Objectives:**

Carotid artery geometry influences blood flow disturbances and is thus an important risk factor for carotid atherosclerosis. Extracellular matrix (ECM) and yes-associated protein (YAP) expression may play essential roles in the pathophysiology of carotid artery stenosis, but the effect of blood flow disturbances of carotid bifurcation location on the ECM is unknown. We hypothesized that carotid artery anatomy and geometry are independently associated with the ECM and YAP expression.

**Methods:**

In this cross-sectional study, 193 patients were divided into two groups: an asymptomatic group (*n* = 111) and a symptomatic group (*n* = 82), symptomatic patients presenting with ischemic attack, amaurosis fugax, or minor non-disabling stroke. For all subjects before surgery, carotid bifurcation angle and internal artery angle were measured with computed tomography angiography (CTA), and laminar shear stress was measured with ultrasonography. After surgery, pathology of all plaque specimens was analyzed using hematoxylin and eosin (HE) staining and Movat special staining. Immunohistochemistry was performed to detect expression of YAP in a subset of 30 specimens.

**Results:**

Symptomatic patients had increased carotid bifurcation angle and laminar shear stress compared to asymptomatic patients (*P* < 0.05), although asymptomatic patients had increased internal carotid angle compared to symptomatic patients (*P* < 0.001). Relative higher bifurcation angles were correlated with increased carotid bifurcation, decreased internal angle, and decreased laminar shear stress. For each change in intervertebral space or one-third of vertebral body height, carotid bifurcation angle changed 4.76°, internal carotid angle changed 6.91°, and laminar shear stress changed 0.57 dynes/cm^2^. Pathology showed that average fibrous cap thickness and average narrowest fibrous cap thickness were greater in asymptomatic patients than symptomatic patients (*P* < 0.05). Expression of proteoglycan and YAP protein in symptomatic patients was higher than in asymptomatic patients (*P* < 0.001), while collagen expression was lower in symptomatic patients than asymptomatic patients (*P* < 0.05).

**Conclusion:**

Geometry of the carotid artery and position relative to cervical spine might be associated with ECM and YAP protein expression, which could contribute to carotid artery stenosis.

## Introduction

Carotid atherosclerosis represents a significant cause of ischemic stroke (Hollander et al., 2002). With rapid economic development and population aging in China, stroke has become the number one cause of death (Liu et al., 2007; Gao et al., 2018). Stroke-related morbidity and mortality now have an enormous socioeconomic impact throughout the country. However, traditional risk factors such as hypertension, diabetes, and hyperlipidemia do not fully explain the occurrence and development of carotid atherosclerosis (Pfenniger et al., 2012).

Carotid atherosclerotic plaques mainly occur in or near arterial bifurcations and bends (Friedman et al., 1983). Local hemodynamic forces, specifically geometry and correlated local blood flow shear stress, can help explain frequent atherosclerosis in carotid bifurcations (Lee et al., 2008).

The ECM is a vital corporal component and is responsible for proper function of various organs. In the cardiovascular system, the ECM participates in and controls many essential functions of the heart and vessels, keeping structural integrity of the cardiovascular network, generating a framework for cell attachment, moderating cell adhesion and cell–cell connections, affecting cell survival and apoptosis, controlling diastolic stiffness, and adjusting for inflammation, vascular injury, and development (Chistiakov et al., 2013). ECM changes in quantity and quality thus may have a relationship with vessel geometry and could affect clinical outcomes.

Yes-associated protein plays an important role in the Hippo pathway as a key functional effector (Zhao et al., 2011). Many studies have found that the Hippo/YAP pathway plays an essential role in organ size control, tissue homeostasis, and cancer, as well as development of the cardiovascular system and maintenance of vessel homeostasis (He et al., 2018). YAP also functions as a structural and mechanical sensor of the microenvironment and can regulate expression of proteins that contribute to tissue stiffness.

Mechanical forces such as heart pumping, fluid shear stress, pressure, and tensional forces in the skeletal system influence cells by controlling gene expression (Panciera et al., 2017). In addition, carotid plaque pathology, such as calcification and osteogenesis (Fisher et al., 2005; Finn et al., 2010; Cocks et al., 2017), can produce mechanical forces.

Current evidence indicates a relationship between carotid geometry and atherosclerosis in occidental countries (Giannattasio et al., 2001; Bijari et al., 2014; Huang et al., 2016), but few studies have examined Chinese populations. Because of food and cultural differences, western results cannot fully be applied to an Asian population. It is unclear whether local lumen geometry contributes to the ECM and to carotid plaque vulnerability. Therefore, the objective of this study was to test whether carotid geometry affects clinical manifestation of atherosclerosis and to identify the relationship of local geometry with ECM and YAP expression.

## Materials and Methods

### Clinical Characteristic of Patients

A total of 193 patients (155 males and 38 females) with carotid stenosis who underwent CEA from January 2016 to December 2017 were included in this study. This research was carried out in accordance with recommendations of the China-Japan Friendship Hospital Ethical Committee. All the patients who received CEA signed informed consent with the Declaration of Helsinki. Patients were informed of the surgical procedures, alternative management, and necessary pathological research.

According to operation criteria, patients were divided into an asymptomatic group and a symptomatic group. Asymptomatic patients had no clinical symptoms before surgery but showed ≥70% stenosis on ultrasonography, or ≥80% on CTA or MRA if ultrasonography stenosis was 50–69%, upon physical examination (Gurm et al., 2008; Redgrave et al., 2008; Rosenfield et al., 2016). Symptomatic patients had a transient ischemic attack, amaurosis fugax, or minor non-disabling stroke including the ipsilateral carotid artery within 6 months before surgery (Grotta, 2013; Brott et al., 2016), as well as ≥70% stenosis on ultrasonography, or ≥70% on CTA or MRA if ultrasonography stenosis was 50–69%, upon physical examination.

Demographic characteristics, medical and reproductive history, drug use, and behavioral habits of subjects were also collected. Blood samples for blood viscosity were collected. Anthropometric data were also collected, including blood pressure, weight, height, and BMI.

### Carotid Endarterectomy and Tissue Processing

Specimens were collected from the same region of the internal carotid artery, 2–4 mm above the bifurcation. Carotid artery segments were fixed in 4% phosphate-buffered formalin for at least 48 h and decalcified in 10% ethylenediaminetetraacetic acid for 72 h. Cross-sectioning was performed at 1-cm intervals, and resulting sections were embedded in paraffin. Each subject had about seven paraffin blocks (1351 paraffin blocks total). All tissue sections were cut at 4-μm thickness, mounted on glass slides, and stained with hematoxylin and eosin (HE) or Movat special staining. Movat staining highlights different tissue components in different colors, including collagen in yellow and proteoglycan in blue. On some specimens (30 patients), we also performed immunohistochemistry using a YAP antibody (1:200; Santa-Cruz Biotechnology).

### Pathology and Immunohistochemistry

All plaque specimens were studied pathologically, including morphology of plaques, size of necrotic lipid core, crystal of cholesterol, continuity, and thickness of fibrous cap. Thickness of the fibrous cap and area of lipid-rich necrotic core were measured under a microscope. Carotid plaques were classified into stable, unstable, and VPs based on fibrous cap thickness and lipid-rich necrotic core area. Lesions displaying a thin fibrous cap (≤165 μm) with infiltrated macrophages and a necrotic core area of ≤40% containing several cholesterol clefs were considered VPs (Finn et al., 2010; Butcovan et al., 2016). USPs were defined as having a fibrous cap thickness of <65 μm and a necrotic lipid core area of >40% (Redgrave et al., 2006; Butcovan et al., 2016). Other lesions were defined as SPs (Virmani et al., 2006; Hellings et al., 2010). Each slide was examined under a microscope, and lipid core area and fibrous cap thickness were measured using Image-Pro Plus 5.1 software. Densitometry analysis was used to quantify results, expressed as average IOD per unit area.

### Computed Tomography Angiography Markers

**FIGURE 1:**
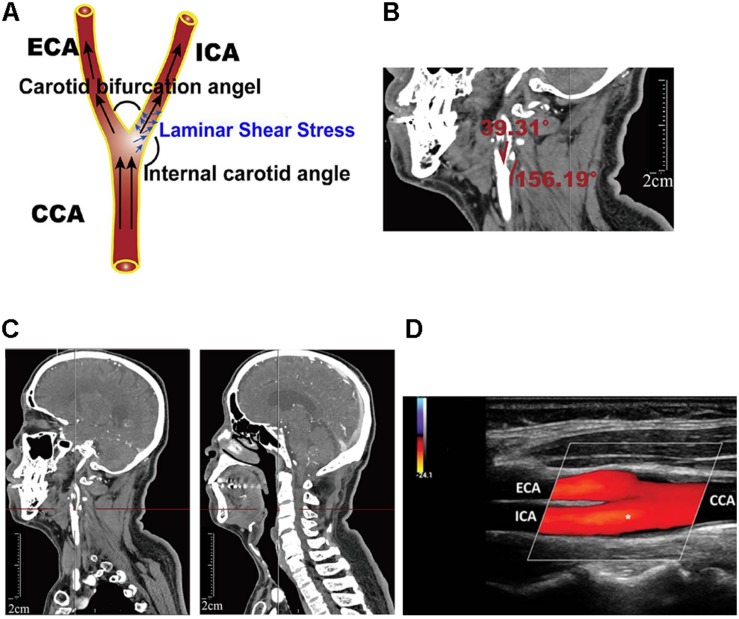
Definition of geometric parameters. **(A)** Schematic representation of geometric parameters, including carotid bifurcation angle, internal carotid angle, and related laminar shear stress, angle of the internal carotid angle with respect to the common carotid artery, hereafter termed “internal carotid angle.” **(B)** Computed tomography angiography (CTA) image illustrating the method of measuring carotid bifurcation angle and internal carotid angle (red line). **(C)** CTA image illustrating measurement of carotid bifurcation angle height relative to the cervical spine. **(D)** Ultrasonography illustrating measurement of blood flow rate and the diameter of the blood vessels.

Computed tomography angiography markers included the common carotid artery bifurcation angle and relative height to the cervical spine as well as internal carotid artery angle. All measurements were obtained using the submillimeter tool on a picture archiving and communication systems (PACS) workstation. To measure angle degree, common, internal, and external angles were visualized in the sagittal plane using PACS to measure vessel border, carotid artery bifurcation angle, and internal carotid artery angle, respectively. Methods of carotid bifurcation angle and internal carotid angle measurement are shown in Figures 1A,B.

The height of carotid bifurcation angle relative to cervical vertebrae was expressed in unit from 1 (high) to 12 (low) (Supplementary Figure S1). For detailed measurement on PACS, carotid bifurcation angle was found and the cursor was moved to the angle. Gradually moving to the middle center sagittal plane, cursor position remained unchanged when moving the cursor, using the cursor’s position as an indicator of cervical vertebrae height. Using this cervical vertebrae height as an indirect indicator of bifurcation angle, relative height of carotid bifurcation angle was measured as shown in Figure 1C.

### Measurement of Blood Viscosity and Laminar Shear Stress

Fasting venous blood samples (6 ml) were collected into heparinized tubes during the first 24 h of hospital admission, and blood viscosity was measured within 2 h using an LS40 blood rheometer according to the manufacturer’s protocol (Contraves Ltd., Switzerland).

Blood flowing in the blood vessels can produce different shear stresses, including laminar shear stress and oscillatory shear stress, which both can affect vessel pathophysiology. Our study showed laminar shear stress to the vessel wall, which we calculated for the carotid artery (τm, in dynes/cm^2^) using the following formula:

$$\tau \,m=\frac{\text{η}\times 4\,\text{Vm}}{\text{Dr}}$$

where η represents the blood viscosity (mPa⋅s), Vm is the average blood velocity (cm/s) (obtained through ultrasonography), and Dr is the arterial diameter in the diastolic period (mm) (obtained through ultrasonography). The position of ultrasonography measurements was mainly located near the carotid bifurcation, with the measuring plane simultaneously visualizing the common carotid artery, external carotid artery, and internal carotid artery. Asterisk (internal carotid artery, ICA) indicated where the blood flow rate and the diameter of the blood vessels was measured (Figure 1D).

### Statistical Analysis

All statistical analyses were performed using the SPSS statistical package (version 21.0; SPSS Inc., Chicago, IL, United States). Continuous data are presented as mean ± standard error of mean (SEM). Comparisons between groups were performed by independent sample *t*-tests for normally distributed data with homogeneous variances or with the non-parametric Mann–Whitney *U*-test. Correlations between carotid bifurcation angle and other parameters were explored using the Pearson correlation coefficient for normally distributed data or Spearman rank correlation coefficient for non-normally distributed or ranked data. *P*-values were two-sided, and *P* < 0.05 was considered statistically significant. Statistical charts were generated using GraphPad Prism 7.0 software (GraphPad Software Inc., La Jolla, CA, United States).

## Results

### Demographic and Clinical Characteristics

Demographic and baseline clinical characteristics are presented in Table 1. Of the 111 asymptomatic and 82 symptomatic patients in the study, no significant difference in age, gender distribution, or BMI was found between groups. 113 left carotids and 80 right carotids were measured, including 62 left carotids and 49 right carotids in asymptomatic group, 51 left carotids and 31 right carotids in symptomatic group. Systolic and diastolic blood pressure were significantly higher in the symptomatic group than the asymptomatic group (*P* < 0.001). Incidence of previous cardiovascular disease and cerebral vascular disease were also significantly higher in the symptomatic group than the asymptomatic group (*P* < 0.001). Further, the proportion of current smokers was significantly higher in the symptomatic group than the asymptomatic group (*P* < 0.001).

**TABLE 1:** Demographic and baseline characteristics of study population.

	Asymptomatic	Symptomatic	
Characteristics	(*N* = 111)	(*N* = 82)	*P-*value
Male sex	89 (80.18)	66 (80.49)	>0.99
Age (years)	65.87 ± 9.42	66.34 ± 8.01	0.91
BMI (kg/m^2^)	24.86 ± 2.93	25.61 ± 3.17	0.18
Systolic (mm Hg)	129.76 ± 1.04	143.72 ± 1.38	<0.001
Diastolic (mm Hg)	78.92 ± 0.66	86.17 ± 0.62	<0.001
**Risk factors**			
Smoking	41 (36.94)	57 (69.51)	<0.001
Hypertension	92 (82.88)	68 (82.93)	>0.99
Diabetes	38 (34.23)	28 (34.15)	>0.99
Dyslipidemia	95 (85.59)	70 (85.37)	>0.99
Previous cardiovascular disease	31 (27.93)	47 (57.32)	<0.001
Previous cerebral vascular disease	27 (24.32)	37 (45.12)	<0.001

*Data are presented as means ± standard error of mean (SEM) for age, body mass index (BMI), systolic and diastolic pressure, number of male subjects, smoking, hypertension, diabetes, dyslipidemia, previous cardiovascular disease, and previous cerebral vascular disease (%).*

### Carotid Bifurcation Angle and Internal Carotid Angle

**FIGURE 2:**
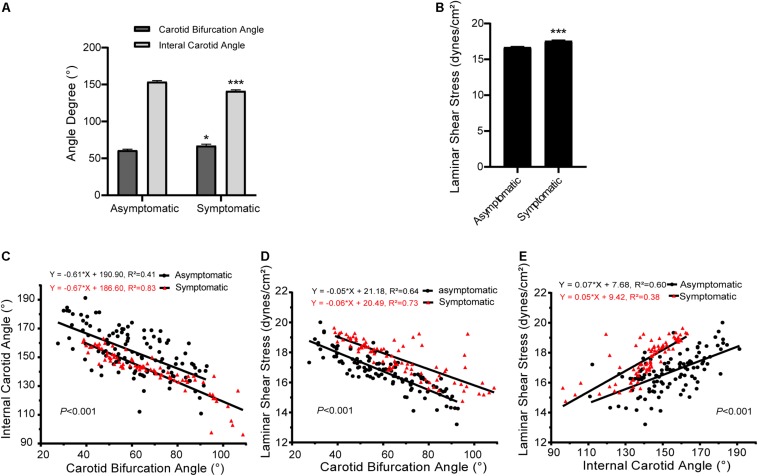
Carotid bifurcation, internal carotid angle, and laminar shear stress in asymptomatic and symptomatic groups. **(A)** Comparison of carotid bifurcation angle and internal carotid angle. **(B)** Comparison of laminar shear stress. **(C)** Correlation of carotid bifurcation angle and internal carotid angle, interaction between asymptomatic and symptomatic groups. **(D)** Correlation of carotid bifurcation angle and laminar shear stress, interaction between asymptomatic and symptomatic groups. **(E)** Correlation of internal carotid angle and laminar shear stress, interaction between asymptomatic and symptomatic groups. Error bars represent standard error of mean (SEM). ^∗^*P* < 0.05, ^∗∗∗^*P* < 0.001.

Detailed data of carotid bifurcation angle and internal carotid angle are shown in Figure 2A and Table 2. Average carotid bifurcation angle was 60.87 ± 1.62° in the asymptomatic group and 67.11 ± 2.08° in the symptomatic group. Average internal carotid angle was 153.80 ± 1.54° in the asymptomatic group and 141.30 ± 1.54° in the symptomatic group. Both of these measures were significantly different between groups. Mean carotid artery blood flow laminar shear stress was 16.69 ± 0.12 dynes/cm^2^ in the asymptomatic group and 17.57 ± 0.14 dynes/cm^2^ in the symptomatic group, also a significant difference (Figure 2B and Table 2).

**TABLE 2:** Comparison of carotid bifurcation, internal carotid angle, and laminar shear stress.

	Asymptomatic	Symptomatic	
Characteristics	(*N* = 111)	(*N* = 82)	*P-*value
Carotid bifurcation angle (°)	60.87 ± 1.62	67.11 ± 2.08	0.02
Internal carotid angle (°)	153.80 ± 1.54	141.30 ± 1.54	<0.001
Laminar shear stress (dynes/cm^2^)	16.69 ± 0.12	17.57 ± 0.14	<0.001

*Data are presented as means ± standard error of mean (SEM) for carotid bifurcation, internal carotid angle, and laminar shear stress.*

Interactions of carotid bifurcation angle, internal carotid angle, and laminar shear stress between groups are shown in Figures 2C–E. The slopes of these interactions significantly differed between asymptomatic and symptomatic groups (*P* < 0.001). Carotid bifurcation angle and internal carotid angle as well as carotid bifurcation angle and laminar shear stress had negative linear correlations. However, internal carotid angle positively correlated with laminar shear stress.

Relative height of the carotid bifurcation angle approximated the frequency of a Gaussian distribution and was classified into three groups: high, normal, or low (Supplementary Figure S1A and Table 3). Normal carotid bifurcation angles were most often located at the level of the superior third of the 4th cervical vertebra; high carotid bifurcation angles were commonly at the level of the superior third or inferior third of vertebra 3, whereas low carotid bifurcation angles were usually found at the level of the middle third of the 5th cervical vertebra. Average carotid bifurcation angle position height was 5.77 (SEM = 0.25) in the asymptomatic group and 5.44 (SEM = 0.25) in the symptomatic group (*P* > 0.05). Average carotid bifurcation angle position height in the asymptomatic group was located at the superior third of the 4th cervical vertebra, whereas it was located at three and four intervertebral discs in the symptomatic group.

**TABLE 3:** Distribution of carotid bifurcation height and height classification.

	Numbers of bifurcation		
Bifurcation height				
(relative to cervical	Asymptomatic	Symptomatic	Height
vertebra)	(*N* = 111)	(*N* = 82)	classification
1	5 (4.50)	4 (4.88)	High bifurcation
2	8 (7.21)	6 (7.32)	
3	10 (9.01)	8 (9.76)	
4	13 (11.71)	10 (12.20)	Middle bifurcation
5	15 (13.51)	12 (14.63)	
6	19 (17.12)	15 (18.29)	
7	14 (12.61)	11 (13.41)	
8	10 (9.01)	8 (9.76)	
9	7 (6.31)	5 (6.10)	Low bifurcation
10	5 (4.50)	3 (3.66)	
11	3 (2.70)	0	
12	2 (1.80)	0	
Average height	5.77	5.44	*P* > 0.05

*The height of carotid bifurcation angle relative to cervical vertebrae was expressed in unit from 1 (high) to 12 (low). Data are presented as values (%). Numeral 1, vertebra 2, 3 intervertebral disk; Numeral 2, superior one-third of vertebra 3; Numeral 3, middle one-third of vertebra 3; Numeral 4, inferior one-third of vertebra 3; Numeral 5, vertebra 3, 4 intervertebral disk; Numeral 6, superior one-third of vertebra 4; Numeral 7, middle one-third of vertebra 4; Numeral 8, inferior 1/3 of vertebra 4; Numeral 9, vertebra 4, 5 intervertebral disk; Numeral 10, superior one-third of vertebra 5; Numeral 11, middle one-third of vertebra 5; Numeral 12, inferior one-third of vertebra 5.*

Further, carotid bifurcation angle was related to its position and height, with a higher position corresponding to a larger carotid bifurcation angle. With regard to internal carotid angle and laminar shear stress, a higher position corresponded with a smaller internal carotid angle and laminar shear stress, with similar trends in asymptomatic and symptomatic groups (Supplementary Figure S1B and Table 4). For each change in intervertebral space or third of vertebral body height, total average carotid bifurcation angle changed 4.76°, total average internal carotid angle changed 6.91°, and total average laminar shear stress changed 0.57 dynes/cm^2^.

**TABLE 4:** Relationship between height of the carotid bifurcation angle, internal carotid angle, and laminar shear stress.

	Average bifurcation angle (°)		Average internal angle (°)		Laminar shear stress (dynes/cm^2^)	
Height	Asymptomatic	Symptomatic	Asymptomatic	Symptomatic	Asymptomatic	Symptomatic
1	89.08 ± 1.61	102.80 ± 2.89	131.60 ± 3.85	100.70 ± 2.23	14.29 ± 0.04	15.40 ± 0.27
2	85.93 ± 1.57	98.65 ± 1.92	139.40 ± 4.89	122.00 ± 1.60	15.23 ± 0.08	16.15 ± 0.63
3	78.02 ± 2.45	87.88 ± 2.06	144.60 ± 2.55	133.10 ± 1.80	15.41 ± 0.29	16.46 ± 0.26
4	75.02 ± 1.71	79.02 ± 1.39	147.30 ± 3.36	135.10 ± 1.06	15.88 ± 0.18	16.84 ± 0.37
5	65.11 ± 1.55	68.17 ± 1.12	149.40 ± 3.32	140.90 ± 0.56	16.43 ± 0.10	17.23 ± 0.17
6	56.22 ± 1.38	59.61 ± 1.16	152.10 ± 3.33	144.90 ± 0.46	16.91 ± 0.08	17.91 ± 0.13
7	51.27 ± 1.28	52.37 ± 1.71	156.20 ± 2.60	150.30 ± 0.51	17.20 ± 0.11	18.45 ± 0.07
8	46.13 ± 3.06	47.22 ± 1.51	163.50 ± 3.80	156.30 ± 1.67	17.50 ± 0.19	18.83 ± 0.12
9	42.13 ± 2.71	43.22 ± 2.11	171.40 ± 2.32	156.80 ± 0.89	18.01 ± 0.19	18.86 ± 0.12
10	36.25 ± 1.50	41.65 ± 2.95	176.00 ± 6.25	162.10 ± 0.74	18.59 ± 0.19	19.19 ± 0.12
11	33.98 ± 3.23	/	181.00 ± 1.39	/	19.42 ± 0.11	/
12	33.25 ± 0.81	/	181.10 ± 1.04	/	19.71 ± 0.30	/

*Data are presented as means ± SEM (standard error of mean) for carotid bifurcation, internal carotid angle, and laminar shear stress.*

### Pathology and Immunohistology

**FIGURE 3:**
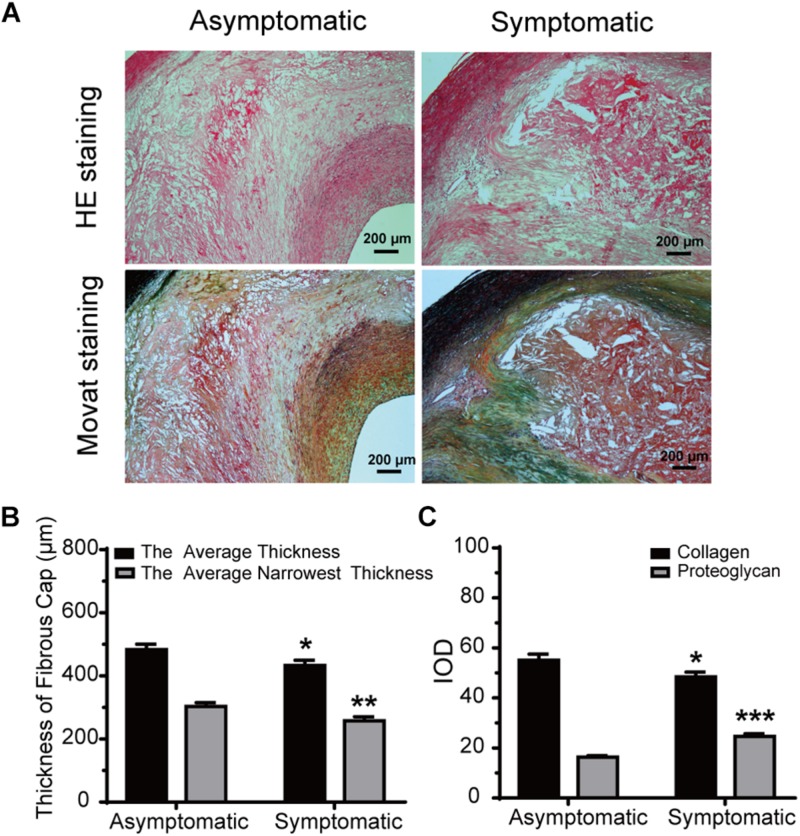
Morphometric analysis data for HE and Movat staining. **(A)** Representative HE and Movat-stained sections of carotid atherosclerotic plaque in asymptomatic and symptomatic groups (bar = 200 μm). **(B)** Average fibrous cap thickness and narrowest thickness of fibrous cap thickness in asymptomatic and symptomatic groups. **(C)** Quantification of collagen and proteoglycan in both groups. Error bars represent standard error of mean (SEM). ^∗^*P* < 0.05, ^∗∗^*P* < 0.01, ^∗∗∗^*P* < 0.001.

Hematoxylin and eosin staining and Movat staining were used to characterize the plaques, which were divided into three groups according to pathological definitions. Movat staining indicated that collagen was more extensively distributed in the fibrous cap in the asymptomatic group than the symptomatic group, while proteoglycan was more distributed in the thin fibrous cap close to the calcification area and larger lipid-rich necrotic core in the symptomatic group (Figure 3A). Thickness of the fibrous cap significantly differed between asymptomatic and symptomatic groups – average fibrous cap thickness was 482.70 ± 17.70 μm in the asymptomatic group and 432.60 ± 16.61 μm in the symptomatic group (*P* < 0.05). Narrowest thickness of the fibrous cap also significantly differed between groups, with 302.70 ± 12.31 μm in the asymptomatic group and 257.60 ± 12.62 μm in the symptomatic group (*P* < 0.01) (Figure 3B and Table 5).

**TABLE 5:** Pathologic characteristic of 193 plaques (111 asymptomatic, 82 symptomatic) and immunohistochemistry for 30 patients (15 asymptomatic, 15 symptomatic).

	Asymptomatic	Symptomatic	
Characteristics	(*N* = 111)	(*N* = 82)	*P-*value
Type of plaques			
Stable	81 (72.97%)	14 (17.07%)	<0.001
Vulnerable plaque	19 (17.12%)	27 (32.93%)	>0.99
Unstable plaque	9 (8.11%)	49 (59.76%)	<0.001
Thickness of fibrous cap			
Average thickness (μm)	482.70 ± 17.70	432.60 ± 16.61	0.04
Average narrowest thickness	302.70 ± 12.31	257.60 ± 12.62	0.01
(μm)			
Extracellular matrix			
Collagen (IOD)	55.11 ± 2.37	48.39 ± 1.39	0.04
Proteoglycan (IOD)	16.32 ± 0.64	24.56 ± 1.13	<0.001
Immunohistochemistry	N = 15	N = 15	
YAP (IOD)	3153.03 ± 178.69	3647.17 ± 229.45	<0.001

*Data are presented as means ± SEM (standard error of mean) for fibrous cap thickness and narrowest thickness for collagen and proteoglycan or as numbers (%) of stable, vulnerable, and unstable plaque. IOD, integrated optical density; YAP, yes-associated protein.*

With respect to ECM, collagen IOD was significantly increased in the asymptomatic group (55.11 ± 2.37) compared to the symptomatic group (48.39 ± 1.93) (*P* < 0.05). However, proteoglycan IOD was significantly increased in the symptomatic group (24.56 ± 1.13) compared to the asymptomatic group (16.32 ± 0.64) (*P* < 0.001) (Figure 3C and Table 5).

### Correlation Between Carotid Geometry and Plaque Pathology

**FIGURE 4:**
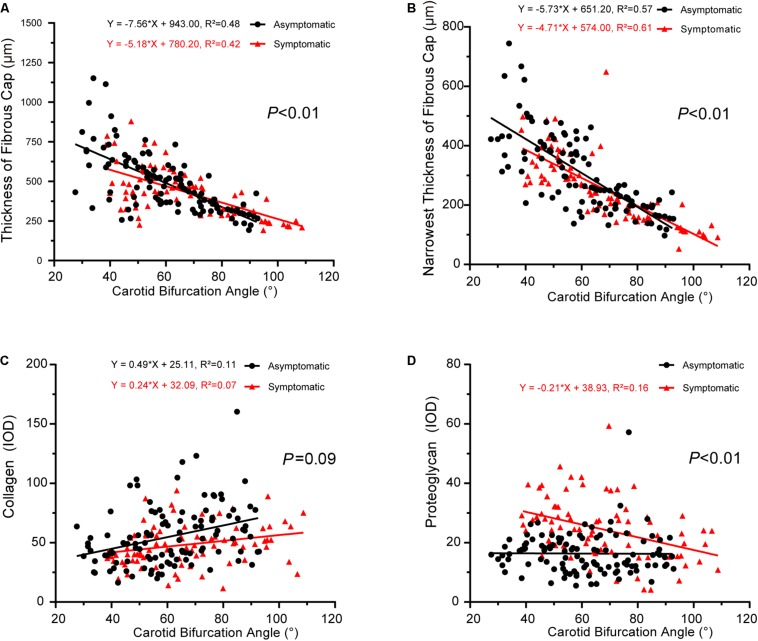
Correlation between carotid bifurcation angle and plaque pathology. **(A)** Linear regression model of fibrous cap thickness and carotid bifurcation angle (asymptomatic: *P* < 0.001, *R*^2^ = 0.48; symptomatic: *P* < 0.001, *R*^2^ = 0.42); interaction between two groups had statistical significance (*P* < 0.01). **(B)** Linear regression model of narrowest thickness of fibrous cap and carotid bifurcation angle (asymptomatic: *P* < 0.001, *R*^2^ = 0.57; symptomatic: *P* < 0.001, *R*^2^ = 0.61); interaction between two groups had statistical significance (*P* < 0.01). **(C)** Linear regression model of collagen and carotid bifurcation angle (asymptomatic: *P* < 0.001, *R*^2^ = 0.11; symptomatic: *P* < 0.001, *R*^2^ = 0.07); interaction between two groups had no statistical significance (*P* = 0.09). **(D)** Linear regression model of proteoglycan and carotid bifurcation angle (asymptomatic: *P* > 0.05; symptomatic *P* < 0.001, *R*^2^ = 0.16); interaction between two groups had statistical significance (*P* < 0.01).

We next explored the correlation between carotid geometry (carotid bifurcation angle, internal carotid angle, laminar shear stress) and pathology (collagen, proteoglycan) in asymptomatic and symptomatic groups. The slopes of interactions between carotid bifurcation angle and pathology significantly differed between asymptomatic and symptomatic groups, with the exception of collagen (Figure 4). Carotid bifurcation angle was negatively correlated with fibrous cap thickness in both groups (asymptomatic: *P* < 0.001, *R*^2^ = 0.48; symptomatic: *P* < 0.001, *R*^2^ = 0.42) (Figure 4A), and carotid bifurcation angle negatively correlated with the narrowest thickness of the fibrous cap in both groups (asymptomatic: *P* < 0.001, *R*^2^ = 0.57; symptomatic: *P* < 0.001, *R*^2^ = 0.61) (Figure 4B). Carotid bifurcation angle positively correlated with collagen in asymptomatic group (asymptomatic: *P* < 0.001, *R*^2^ = 0.11; symptomatic: *P* < 0.001, *R*^2^ = 0.07) (Figure 4C). Carotid bifurcation angle negatively correlated with proteoglycan in the symptomatic group (*P* < 0.001, *R*^2^ = 0.16), but no correlation was found in the asymptomatic group (*P* > 0.05) (Figure 4D).

**FIGURE 5:**
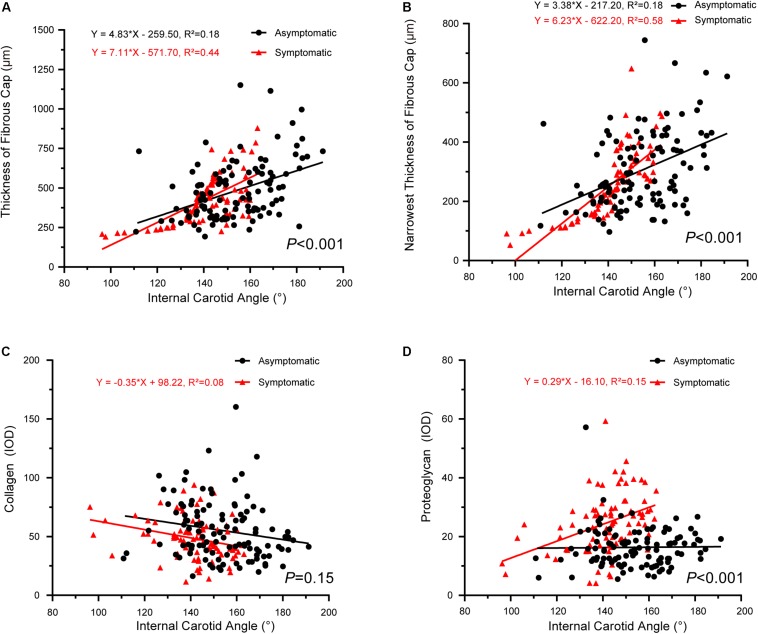
Correlation between internal carotid angle and plaque pathology. **(A)** Linear regression model of fibrous cap thickness and internal carotid angle (asymptomatic: *P* < 0.001, *R*^2^ = 0.18; symptomatic: *P* < 0.001, *R*^2^ = 0.44); interaction between two groups had statistical significance (*P* < 0.001). **(B)** Linear regression model of narrowest thickness of fibrous cap and internal carotid angle (asymptomatic: *P* < 0.001, *R*^2^ = 0.18; symptomatic: *P* < 0.001, *R*^2^ = 0.58); interaction between two groups had statistical significance (*P* < 0.001). **(C)** Linear regression model of thickness of collagen and internal carotid angle (asymptomatic: *P* > 0.05; symptomatic: *P* < 0.001, *R*^2^ = 0.08); interaction between two groups had no statistical significance (*P* = 0.15). **(D)** Linear regression model of proteoglycan and internal carotid angle (asymptomatic: *P* > 0.05; symptomatic: *P* < 0.01, *R*^2^ = 0.15); interaction between two groups had statistical significance (*P* < 0.001).

The correlations between internal carotid angle and pathology also had significantly different slopes between asymptomatic and symptomatic groups, with the exception of proteoglycan/collagen (Figure 5). Internal carotid angle had a positive correlation with fibrous cap thickness in both groups (asymptomatic: *P* < 0.001, *R*^2^ = 0.18; symptomatic: *P* < 0.001, *R*^2^ = 0.44) (Figure 5A). Internal carotid angle also positively correlated with the narrowest thickness of the fibrous cap (asymptomatic: *P* < 0.001, *R*^2^ = 0.18; symptomatic: *P* < 0.001, *R*^2^ = 0.58) (Figure 5B). However, internal carotid angle negatively correlated with collagen in the symptomatic group (*P* < 0.001, *R*^2^ = 0.08), but no correlation was found in the asymptomatic group (*P* > 0.05, *R*^2^ = 0.02) (Figure 5C). Internal carotid angle also negatively correlated proteoglycan in the symptomatic group (*P* < 0.01, *R*^2^ = 0.15), but no correlation was found in the asymptomatic group (*P* > 0.05) (Figure 5D).

**FIGURE 6:**
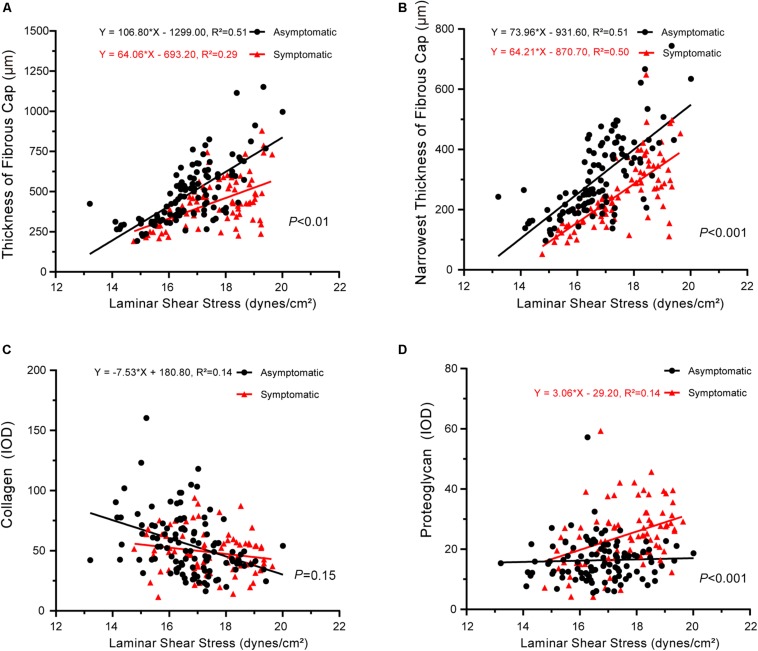
Correlation between laminar shear stress and plaque pathology. **(A)** Linear regression model of fibrous cap thickness and laminar shear stress (asymptomatic: *P* < 0.001, *R*^2^ = 0.51; symptomatic: *P* < 0.001, *R*^2^ = 0.29); interaction between two groups had statistical significance (*P* < 0.01). **(B)** Linear regression model of narrowest thickness of fibrous cap and laminar shear stress (asymptomatic: *P* < 0.001, *R*^2^ = 0.51; symptomatic: *P* < 0.001, *R*^2^ = 0.50); interaction between two groups had statistical significance (*P* < 0.001). **(C)** Linear regression model of collagen and laminar shear stress (asymptomatic: *P* < 0.001, *R*^2^ = 0.14; symptomatic: *P* > 0.05); interaction between two groups had no statistical significance (*P* = 0.15). **(D)** Linear regression model of proteoglycan and laminar shear stress (asymptomatic: *P* > 0.05; symptomatic: *P* < 0.01, *R*^2^ = 0.14); interaction between two groups had statistical significance (*P* < 0.001).

Finally, the slopes between laminar shear stress and pathology also significantly differed between asymptomatic and symptomatic groups, with the exception of collagen (Figure 6). Laminar shear stress was positively correlated with fibrous cap thickness (asymptomatic: *P* < 0.001, *R*^2^ = 0.51; symptomatic: *P* < 0.001, *R*^2^ = 0.29) (Figure 6A). Laminar shear stress also was positively correlated with the narrowest thickness of the fibrous cap (asymptomatic: *P* < 0.001, *R*^2^ = 0.51; symptomatic: *P* < 0.001, *R*^2^ = 0.50) (Figure 6B). However, laminar shear stress negatively correlated with collagen in the asymptomatic group (*P* < 0.001, *R*^2^ = 0.14), but no correlation was found in the symptomatic group (*P* > 0.05) (Figure 6C). Laminar shear stress positively correlated with proteoglycan in the symptomatic group (*P* < 0.01, *R*^2^ = 0.14), but no correlation was found in the asymptomatic group (*P* > 0.05) (Figure 6D).

### Expression of YAP in Carotid Plaques

**FIGURE 7:**
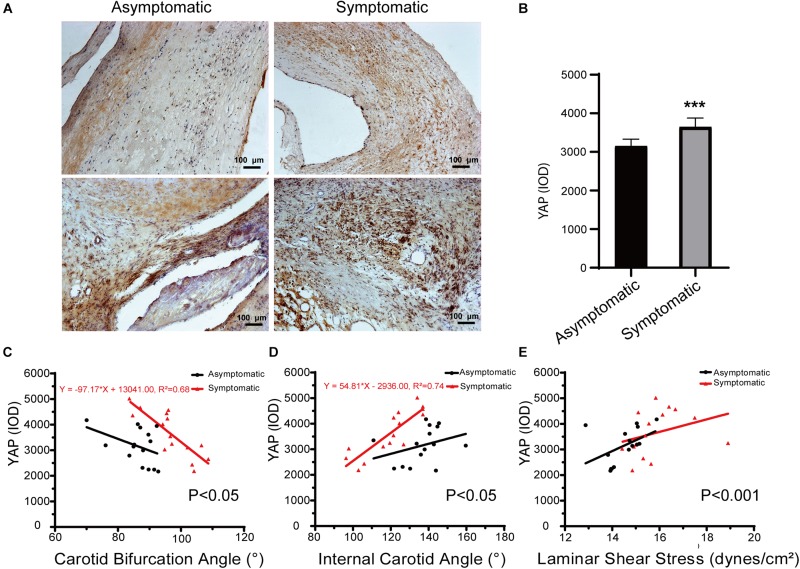
Immunohistochemical staining of YAP in carotid atherosclerotic plaque. **(A)** Representative immunochemical results of yes-associated protein (YAP) expression in asymptomatic patients and symptomatic patients (bar = 100 μm). **(B)** Quantification of IOD of YAP in asymptomatic patients and symptomatic patients. Error bars represent standard error of mean (SEM). ^∗∗∗^*P* < 0.001. **(C)** Linear regression model of carotid bifurcation angle and YAP expression (asymptomatic: *P* > 0.05; symptomatic: *P* < 0.001, *R*^2^ = 0.68); interaction between two groups had statistical significance (*P* < 0.05). **(D)** Linear regression model of internal carotid angle and YAP expression (asymptomatic: *P* > 0.05; symptomatic: *P* < 0.001, *R*^2^ = 0.74); interaction between two groups had statistical significance (*P* < 0.05). **(E)** Linear regression model of laminar shear stress and YAP expression (asymptomatic: *P* > 0.05; symptomatic: *P* > 0.05); interaction between two groups had statistical significance (*P* < 0.001).

In carotid atherosclerotic plaque specimens, YAP is mainly distributed in the area of calcification. In a subset of our samples, YAP expression was significantly higher in the symptomatic group, and these trends were mainly distributed close to angiogenesis, calcification areas, and the tunica media, showing a granular appearance (Figure 7A). Further, YAP had significantly higher distribution in fibrous caps of the symptomatic group than in the asymptomatic group – average YAP IOD was 3647.17 ± 229.45 in the symptomatic group and 3153.03 ± 178.69 in the asymptomatic group (Figure 7B and Table 5) (*P* < 0.001).

Regarding the correlation between carotid geometry and YAP in asymptomatic and symptomatic groups, the slope between carotid bifurcation and YAP was significant (Figure 7C). Carotid bifurcation angle had a linear positive correlation with YAP distribution in symptomatic group (*P* < 0.001, *R*^2^ = 0.68), no correlation in the asymptomatic group (*P* > 0.05). Further, internal carotid angle had a linear positive correlation with YAP distribution in symptomatic group (*P* < 0.001, *R*^2^ = 0.74), but no correlation in the asymptomatic group (*P* > 0.05) (Figure 7D). There had no correlation between laminar shear stress and YAP distribution (asymptomatic: *P* > 0.05; symptomatic: *P* > 0.05) (Figure 7E).

## Discussion

The pathology of carotid artery atherosclerosis has a significant association with the occurrence of stroke. Scientists have long attributed atherosclerosis to hypertension, hyperlipidemia, and diabetes, but these theories cannot fully explain the pathophysiology of atherosclerosis. As research progresses, much attention has focused on geometry of the carotid artery, with atherosclerosis initiating primarily at arterial bends and major branches, especially at the carotid bifurcation (Bijari et al., 2014). Because of altered hemodynamics at the bifurcation, the impact of these factors on the progression of atherosclerosis has been accentuated (Spanos et al., 2017).

The demographics of this study population included more male atherosclerosis patients than females, with a ratio of 4:1. This may be explained by more male smokers than females in current Chinese society. Considering clinical symptoms, 57.5% of patients had no symptoms and 42.5% showed clinical symptoms, including impaired movement, sensation, or language. Increased risk factors (e.g., current smoker, previous cardiovascular disease, previous cerebral vascular disease) have been previously reported in symptomatic patients (West et al., 2015; Niemann et al., 2017; Hoshino et al., 2018), and these traditional risk factors are closely related to carotid atherosclerosis (Redgrave et al., 2006; Virmani et al., 2006). We found that symptomatic patients were more likely to smoke (including present and ever) and have previous cardiovascular or cerebral vascular disease, despite the lack of significant differences in hypertension, diabetes, and dyslipidemia between groups. These risk factors also have been attributed to the pathogenesis of atherosclerosis. However, individuals who undergo CEA already have a high ratio of hypertension, diabetes, and dyslipidemia.

Previous studies repeatedly confirm that carotid bifurcation geometry is related to carotid atherosclerosis (Sitzer et al., 2003; Tuenter et al., 2016; Spanos et al., 2017). Carotid bifurcation geometry is currently considered a pathological marker for carotid atherosclerosis and contributes to plaque abruption, intraplaque hemorrhage, embolism, and other cephalic symptoms (Phan et al., 2012). Mechanisms of carotid atherosclerosis may involve geometry of the carotid artery, which can affect laminar shear stress and thus may accelerate atherosclerosis in combination with traditional risk factors. In agreement, we found that a larger carotid bifurcation angle tended to have a smaller laminar shear stress in both groups, indicating a potential relationship to clinical symptoms. Moreover, relative height of the carotid bifurcation angle to the cervical vertebra as well as carotid bifurcation angle were increased in the symptomatic group compared to the asymptomatic group, suggesting that carotid bifurcation geometry is a pathological marker for carotid atherosclerosis.

Syo et al. (2005) found that relative height of carotid bifurcation angle also is a risk factor for plaque atherogenesis. Carotid bifurcation angle increases 3.34° with each third increase of cervical vertebral body height or intervertebral space height. Our results are in agreement, but we also tested the theory that there is a relationship between the height of carotid bifurcation angle and laminar shear stress. As the height of carotid bifurcation angle increases, laminar shear stress decreases. Although laminar shear stress protects against atherosclerosis, it was increased in the symptomatic group compared to the asymptomatic group, which may be due to higher mean arterial pressure in symptomatic patients causing increased blood velocity. Logistic regression and adjusted analysis to reduce bias caused by confounding factors or interactions showed that mean arterial pressure remained statically significant (*P* < 0.001) (Supplementary Table S1).

Besides carotid bifurcation angle, internal carotid angle is regarded as an essential factor in early atherosclerotic changes. Sitzer et al. (2003) used Duplex scan research to show that the angle of internal carotid angle origin may be an independent risk factor for early atherosclerotic changes at the internal carotid angle bulb. Our results show a negative correlation between carotid bifurcation angle and internal carotid angle. Further, we also showed a positive correlation between laminar shear stress and internal carotid angle, implying that internal carotid angle might be affected by carotid bifurcation angle and that both angles may affect hemodynamics and thus manifest in diverse clinic symptoms.

The arterial wall consists of a highly ordered structure of different cells and ECM. Components of the interstitial matrix synthesized by endothelial and smooth muscle cells play important roles in supporting the integrity and normal metabolism of the arterial wall (Radhakrishnamurthy et al., 1990). Atherosclerosis begins with eruption of the endothelium, for which smooth muscle cells play a crucial role. In the media of the arterial wall, individual smooth muscle cells are encircled with a basement membrane and are embedded in types I, III, and V collagen, fibronectin, and proteoglycans (Adiguzel et al., 2009). After arterial injury and during atherosclerotic plaque development, prominent changes in ECM composition occur – smooth muscle cells transition from a motionless, contractile phenotype to a proliferative, matrix synthetic phenotype.

Collagen is an essential component in atherosclerotic plaques (Duprez et al., 2017). Cheng et al. (2007) found that low shear stress induces atherosclerotic plaque formation in mice by increasing lipid and matrix metalloproteinase content and decreasing vascular smooth muscle and collagen content. Proteoglycans, carbohydrate–protein macromolecules, have various biological functions and undergo many changes in response to diverse stimuli, such as stress, inflammation, and hormonal influences. Further, these changes are generally determined by local cellular activity (Voelker et al., 1990). Therefore, we hypothesize that blood flow laminar shear stress in the arterial wall and carotid bifurcation angle has a role in ECM synthesis. Our results indicate that increasing laminar shear stress could slightly increase proteoglycan volume in plaques, especially in the symptomatic group, showing a positive correlation. Nevertheless, ECM changes could be affected by carotid artery geometry via the influence of hemodynamics, altering the morphology of atherosclerosis and thus contributing to carotid artery stenosis.

Cells can sense their microenvironment not only through soluble signals but also through physical and mechanical forces, such as ECM stiffness or confined adhesiveness (Dupont et al., 2011). Variations of ECM stiffness affect cell shape and greatly impact cell behavior across cell types, including mesenchymal stem cells, muscle stem cells, and endothelial cells (Panciera et al., 2017). Dupont et al. (2011) showed that YAP/TAZ is a sensor and mediator of mechanical cues from the cellular microenvironment. Xu et al. (2016) found that atheroprotective laminar flow force inhibits endothelial YAP activation, which might contribute to laminar flow-mediated endothelial cell quiescence and anti-inflammation. Consistently, we found higher YAP expression in the symptomatic group compared to the asymptomatic group. Expression in the asymptomatic group was close to the tunica media but also had a larger distribution in the thinnest part of the fibrous cap. In the symptomatic group, YAP was primarily located in the area of calcification and at the thinnest part of the fibrous cap, which may be correlated with cap rupture, hemolysis, and symptomatic occurrence. The interaction of YAP and carotid artery geometry had a significant difference between the two groups. However, a strong correlation between YAP and carotid artery geometry was found only in symptomatic patients. The fact that this correlation is inverse with respect to the expected beneficial role of the carotid artery geometry (higher YAP for higher internal carotid angle and for lower carotid bifurcation angle) throws light on the fact that there are maybe other factors, beside carotid geometry, to explain the higher YAP concentration and the higher risk for plaque rupture.

The present study comprehensively assessed correlation between carotid geometry and clinical carotid pathology. To our knowledge, this study is the first to initially confirm that carotid geometry could affect the human ECM. We found that alterations in both carotid bifurcation angle and internal carotid angle influence the ECM, in accordance with previous reports that clinical manifestation might be related to alteration of both angles (Touze et al., 2006; Phan et al., 2012; Huang et al., 2016).

However, this study has some limitations. First, the size of our study population was limited, which may influence generalizability of our conclusions. Second, our population was not balanced in sex, as most patients were male (80.31%). Third, digitally measuring angles with contrast angiography showed that a portion of patients had a proximal curvature, which is a strong and significant predictor of flow disturbances at the carotid bifurcation (Bijari et al., 2014). However, we did not analyze this data, which might partially explain patient differences. Fourth, there are limitations to how we measured data – both angles were obtained via patients’ CTA on the PACS workstation and were inevitably influenced by patient position and respiration as well as radiologist skill, which might introduce perceptual biases. However, to reduce such error, we had two surgeons separately measure carotid bifurcation and carotid internal angles, each independently measured three times, and we used the average as the final result. Finally, due to the cross-sectional design, our results need to be further verified with well-designed clinical research studies with large sample populations.

Taken together, this study provides new evidence that laminar shear stress might be related to carotid bifurcation angle and internal carotid angle as well as relative height of both angles. Further, we found that different angles could affect laminar shear stress, resulting in different ECM volumes. Moreover, these different ECM volumes might result in different clinical manifestations. These findings could provide further clues for the pathophysiology of carotid atherosclerosis and for the search for more specific and effective therapeutic targets.

## Conclusion

Our data suggest that the geometry and height of carotid artery bifurcation angle and carotid internal angle could result in different laminar shear stress. In addition, change in carotid artery bifurcation angle (including size and height) might alter the ECM and YAP expression, producing different clinical manifestations. Further prospective studies in diverse populations are needed to enhance the generalizability of these findings.

## Data Availability Statement

The datasets generated for this study are available on request to the corresponding author.

## Ethics Statement

The studies involving human participants were reviewed and approved by the China-Japan Friendship Hospital Ethics Committee. The patients/participants provided their written informed consent to participate in this study. Written informed consent was obtained from the individual(s) for the publication of any potentially identifiable images or data included in this article.

## Author Contributions

JW, PL, and YC contributed to the conception and design of the study. YC, XL, FW, JK, HZ, ZY, LP, JW, and PL contributed to the analysis and interpretation. YC, XL, FW, JK, ZY, LP, JW, and PL contributed to the data collection. YC and XL contributed to the writing of the article. JW, YC, CS, XL, FW, JK, ZY, LP, and PL provided critical revision of the article. JW, YC, XL, JK, ZY, LP, and PL provided the final approval of the article. YC, XL, FW, and JK contributed to the statistical analysis. JW and PL obtained funding. JW contributed to the overall responsibility.

## Conflict of Interest

The authors declare that the research was conducted in the absence of any commercial or financial relationships that could be construed as a potential conflict of interest.

## Supplementary Material

FIGURE S1 Distribution of carotid bifurcation angle. **(A)** Total distribution of carotid bifurcation angle in different groups. **(B)** Distribution of carotid bifurcation angle, internal carotid angle, and laminar shear stress at different heights in both groups. Error bars represent standard error of mean (SEM).

TABLE S1 Logistic regression of demographic and baseline characteristics of the studying population.

## Abbreviations

BMI: body mass index
CEA: carotid endarterectomy
CTA: computed tomography angiography
ECM: extracellular matrix
IOD: integrated optical density
MRA: magnetic resonance angiography
SP: stable plaque
USP: unstable plaque
VP: vulnerable plaque
YAP: yes-associated protein
