# Supplementary figures and images for: Geometry of the Carotid Artery and Its Association With Pathologic Changes in a Chinese Population
Source: Front Physiol. 2020 Jan 21;10:1628. doi: 10.3389/fphys.2019.01628 (PMC6985580; doi:10.3389/fphys.2019.01628)

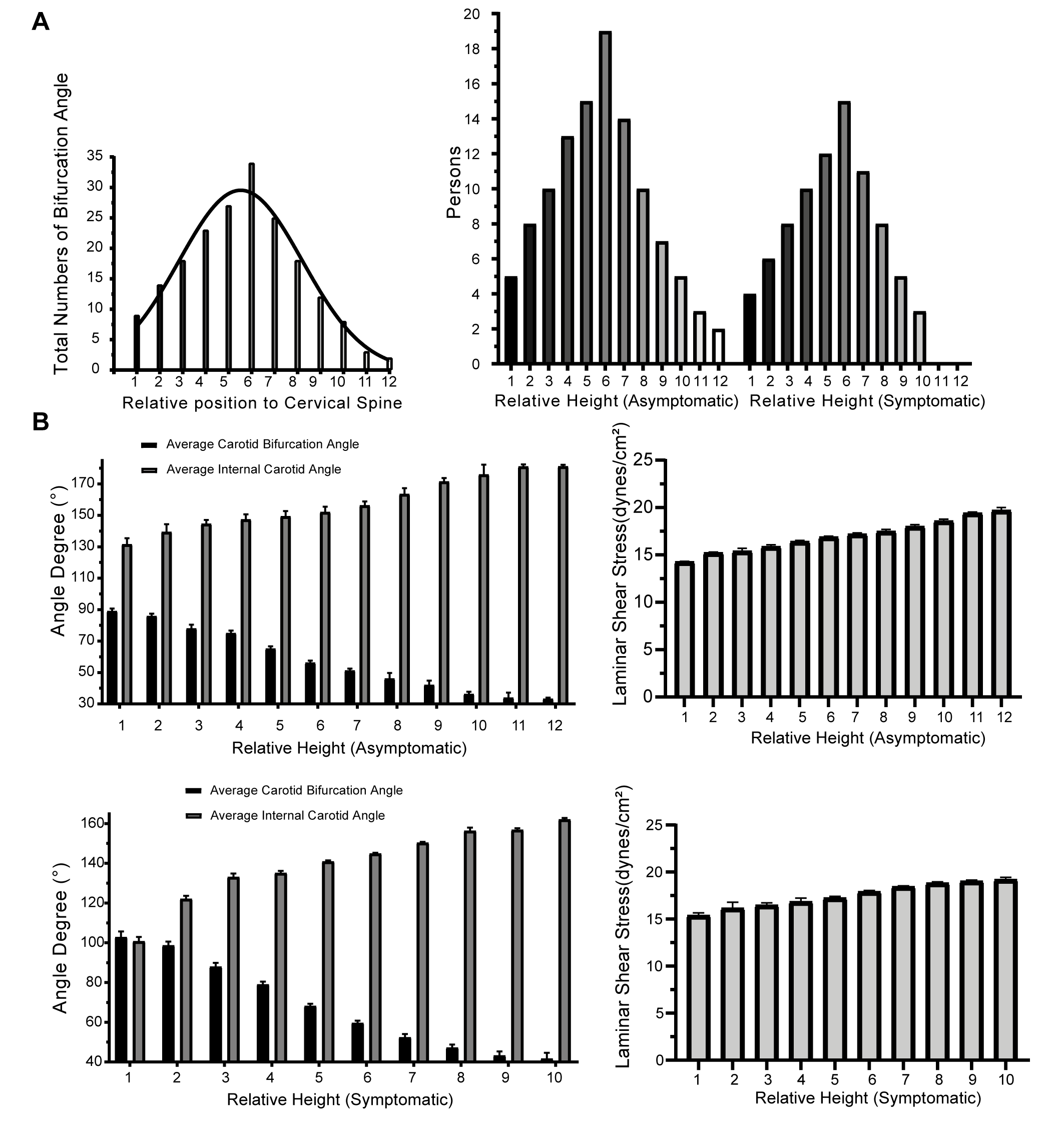

Supplement: FIGURE S1 — Distribution of carotid bifurcation angle. (A) Total distribution of carotid bifurcation angle in different groups. (B) Distribution of carotid bifurcation angle, internal carotid angle, and laminar shear stress at different heights in both groups. Error bars represent standard error of mean (SEM). [file Image_1.TIF]
