# Supplementary material for: Geometry of the Carotid Artery and Its Association With Pathologic Changes in a Chinese Population
Source: Front Physiol. 2020 Jan 21;10:1628. doi: 10.3389/fphys.2019.01628 (PMC6985580; doi:10.3389/fphys.2019.01628)
Supplement: TABLE S1 — Logistic regression of demographic and baseline characteristics of the studying population. [file Table_1.docx]

**Supplementary Table I: Logistic regression of demographic and baseline characteristics of the studying population.**

|  | **Estimate** | **SE** | **Z-value** | **P-value** |
| --- | --- | --- | --- | --- |
| **Intercept** | -126.886 | 30.404 | -4.173 | <0.001 |
| **BMI** | -0.181 | 0.127 | -1.422 | 0.155 |
| **SEX** | 0.664 | 0.906 | 0.732 | 0.464 |
| **Pulse pressure** | -0.040 | 0.033 | -1.210 | 0.226 |
| **Mean arterial pressure** | 0.417 | 0.100 | 4.177 | <0.001 |
| **Smoking** | 0.982 | 0.809 | 1.214 | 0.225 |
| **Dyslipidemia** | -0.748 | 0.762 | -0.981 | 0.326 |
| **Previous event** | -0.141 | 0.761 | -0.186 | 0.853 |
| **AGE** | 0.045 | 0.047 | 0.948 | 0.343 |
| **Carotid bifurcation angle** | 0.286 | 0.082 | 3.497 | <0.001 |
| **Internal carotid angle** | -0.127 | 0.040 | -3.184 | 0.001 |
| **laminar shear stress** | 5.147 | 1.154 | 4.460 | <0.001 |

BMI (body mass index), SE (Std. Error).
